# Expression of programmed death receptor-1 ligand (PD-L1) in human cancer is of prognostic value and associated with macrophage infiltration

**DOI:** 10.7150/jca.99781

**Published:** 2024-11-04

**Authors:** Yu Liu, Qian Li, Gangchi Cao, Huijuan Wei, Chuansong Xue, Jianhao Liu

**Affiliations:** 1 Sanya Hospital of Traditional Chinese Medicine, Hannan, China.; 2 Department of Medical Oncology, Hangzhou Cancer Hospital, Hangzhou, China.; 3 Department of Hematology & Oncology, Shanghai Children's Medical Center, Shanghai Jiao Tong University School of Medicine, National Health Committee Key Laboratory of Pediatric Hematology & Oncology, Shanghai, China.; 4 Department of Clinical Medicine, Sun Yat-sen University School of Medicine, Guangzhou, China.; 5 Guangzhou University of Chinese Medicine, Guangzhou, China.

**Keywords:** PD-L1, macrophage, cancer, immune infiltration, single-cell RNA sequencing

## Abstract

The tumor immune microenvironment is a crucial factor influencing tumor progression, and its molecular mechanisms have become a key topic in immunotherapy research. Programmed death receptor-1 ligand (PD-L1, CD274) is a well-known immunosuppressive molecule that can mediate the immune escape of tumor cells. The aim of this study was to evaluate the significance of PD-L1 in human cancer by integrated bioinformatics analysis. Tumor IMmune Estimation Resource (TIMER), GEPIA, Kaplan-Meier plotter, TISIDB and Tumor Immune Single Cell Hub (TISCH) were used to perform the corresponding analysis. The results showed that PD-L1 was dysregulated in various cancers and was associated with the overall survival of cancer patients, which was associated with macrophage infiltration levels. Moreover, PD-L1 expression showed a significant correlation with macrophages and was universally expressed on tumor-associated macrophages (TAMs). Notably, the expression of PD-L1 on TAMs was found to be correlated with immunotherapy response in certain cancers based on analysis of single-cell RNA sequencing data. In conclusion, PD-L1 plays a significant role in cancer, which may partly be influenced by TAMs.

## Introduction

Cancer morbidity is rapidly rising worldwide, and cancer is among the three leading causes of death in the 21st century 1. Although the pathogenesis of cancer has been gradually revealed and multidisciplinary therapies have been developed, the clinical prognosis of cancer patients remains poor 2. The immune microenvironment around cancer cells plays crucial roles in cancer pathogenesis and is increasingly being investigated 3. Notably, one of the hallmarks of cancer is evasion of immune destruction 4. Expression profile data are being continually uploaded to public databases, providing us with the possibility of using bioinformatic technologies to explore the immune microenvironment in cancer patients 5, 6.

Programmed death receptor-1 ligand (PD-L1, CD274), which serves as a ligand for programmed death receptor-1 (PD-1), is predominantly expressed on the membrane surface of monocytes, macrophages, dendritic cells and various tumor cells 7. Under normal circumstances, the interaction of PD-L1 and PD-1 protects the body from damage caused by excessive inflammation and plays an important role in the treatment of autoimmune diseases 8. When tumors occur, PD-1/PD-L1 signal transduction inhibits the function of lymphocytes and macrophages, and causing apoptosis of lymphocytes, eventually leading to the immune escape of tumor cells. Blocking signal transduction with related anti-PD-1 and/or anti-PD-L1 monoclonal antibodies can restore the immune response, which is a new strategy for tumor immunotherapy, but the clinical effects are quite different in different tumors 9, 10. Additionally, studies have indicated that PD-L1 serves as a crucial prognostic indicator for patients with tumors 11, 12.

Infiltrating macrophages in the tumor microenvironment are related to poor prognosis and are associated with chemotherapy resistance in most cancers 13. Macrophages contribute to tumor progression in various ways: by stimulating angiogenesis, nurturing cancer stem cells, increasing migration, and suppressing antitumor immunity 14. Based on signals received from tumor microenvironment, such as granulocyte-macrophage colony-stimulating factor (GM-CSF), colony-stimulating factor 1 (CSF1), IFN-γ, M0 macrophages polarize into two subtypes of tumor-associated macrophages (TAMs): anti-tumoral M1 and pro-tumoral M2-like TAMs. The phenotype of TAMs has been shown to be strongly correlated with cancer stage and prognosis 10, 15. Multiple studies have found that a treatment scheme targeting macrophages showed excellent therapeutic effect and certain clinical benefits 16-18. Therefore, it is particularly important to identify the key molecules that regulate the function of macrophages in the tumor microenvironment.

In this study, we analyzed the expression and prognostic value of PD-L1 in different types of cancer. Subsequently, we explored the association of PD-L1 expression with macrophage infiltration levels and the marker genes of macrophages in some cancers. Additionally, using single-cell RNA sequencing (scRNA-seq) data, we evaluated the expression of PD-L1 on TAMs and its therapeutic implications.

## Materials and Methods

### TIMER database

The Tumor IMmune Estimation Resource (TIMER) database (http://timer.cistrome.org/) was employed to analyze the expression of PD-L1 in cancers and normal tissues and to estimate the correlations between PD-L1 expression and macrophage infiltration levels 19. Moreover, we explored the outcomes of cancer patients by comprehensively considering macrophage infiltration and PD-L1 expression levels. The expression of PD-L1 was also analyzed with the Gene Expression Profiling Interactive Analysis (GEPIA) database (http://gepia2.cancer-pku.cn/#index) 20. Spearman's correlation analysis to describe the correlation between PD-L1 expression and macrophages. P values less than 0.05 were considered statistically significant.

### Kaplan-Meier plotter

The online Kaplan-Meier plotter database (http://kmplot.com/analysis/) and GEPIA database were adopted to explore the prognostic value of PD-L1 in various cancers 21. Kaplan-Meier plotter includes 21 cancer types, and sources for the databases include GEO, EGA, and TCGA. Kaplan-Meier survival plots were constructed to reveal the association of PD-L1 expression with the overall survival of cancer patients. The log-rank test was used to determine the statistical significance of the correlation. P values less than 0.05 were regarded as statistically significant.

### TISIDB

TISIDB (http://cis.hku.hk/TISIDB) was employed to estimate the relationship between the abundance of TAMs and the expression of PD-L1 22. TISIDB is a user-friendly web portal that has precalculated the associations between genes and immune features for 30 TCGA cancer types. The expression data are first log2(TPM+1) transformed for differential analysis.

### scRNA-seq analysis

Tumor Immune Single Cell Hub (TISCH, http://tisch.comp-genomics.org) was adopted to explore the expression of PD-L1 on TAMs and the corresponding clinical value. This database includes single-cell transcriptomic profiles from nearly 2 million cells from 76 high-quality tumor datasets across 27 cancer types 23.

## Results

### Abnormal expression of PD-L1 in cancer

**Figure 1:**
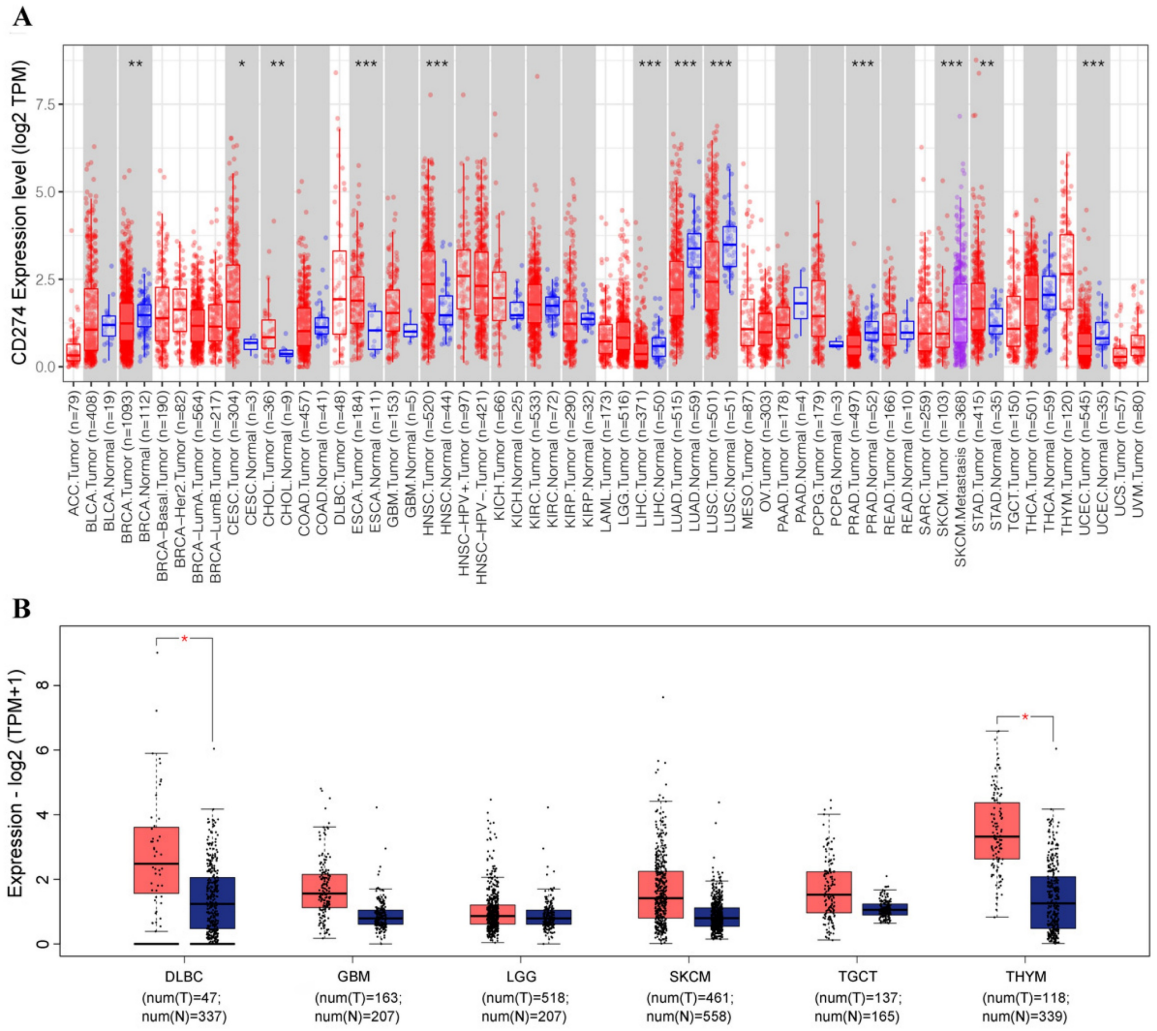
PD-L1 expression was abnormal in various cancers based on TIMER (A) and GEPIA (B) analyses. *p < 0.05, **p < 0.01, ***p < 0.001, asterisks (*) stand for significance levels.

We employed the TIMER database to analyze the expression of PD-L1 in different types of human cancer. PD-L1 levels in cancer tissues and in normal tissues were compared based on a TCGA dataset. As shown in Figure 1A, PD-L1 expression in various cancers was obviously different from that in normal tissues. The expression levels of PD-L1 in the tumor tissues of cervical squamous cell carcinoma (CESC), cholangiocarcinoma (CHOL), esophageal carcinoma (ESCA), head and neck squamous cell carcinoma (HNSC), and stomach adenocarcinoma (STAD) were higher than those in the corresponding control tissues.

The expression of PD-L1 was downregulated in breast invasive carcinoma (BRCA), liver hepatocellular carcinoma (LIHC), lung adenocarcinoma (LUAD), lung squamous cell carcinoma (LUSC), prostate adenocarcinoma (PRAD), and uterine corpus endometrial carcinoma (UCEC).

With the GEPIA database, we further evaluated the expression level of PD-L1 in lymphoid neoplasm diffuse large B-cell lymphoma (DLBC), glioblastoma multiforme (GBM), brain lower grade glioma (LGG), skin cutaneous melanoma (SKCM), testicular germ cell tumor (TGCT), and thymoma (THYM). The results showed that the expression of PD-L1 was significantly upregulated in DLBC and THYM (Figure 1B).

### The prognostic value of PD-L1 is related to macrophages in various cancers

Subsequently, we constructed Kaplan-Meier survival curves to determine the prognostic value of PD-L1 in cancer patients. PD-L1 was found to be significantly associated with overall survival in breast cancer, kidney renal clear cell carcinoma, LIHC, ovarian cancer (OV), pancreatic ductal adenocarcinoma (PAAD), sarcoma, TGCT, THYM, UCEC, LGG and SKCM (Figure S1).

**Figure 2:**
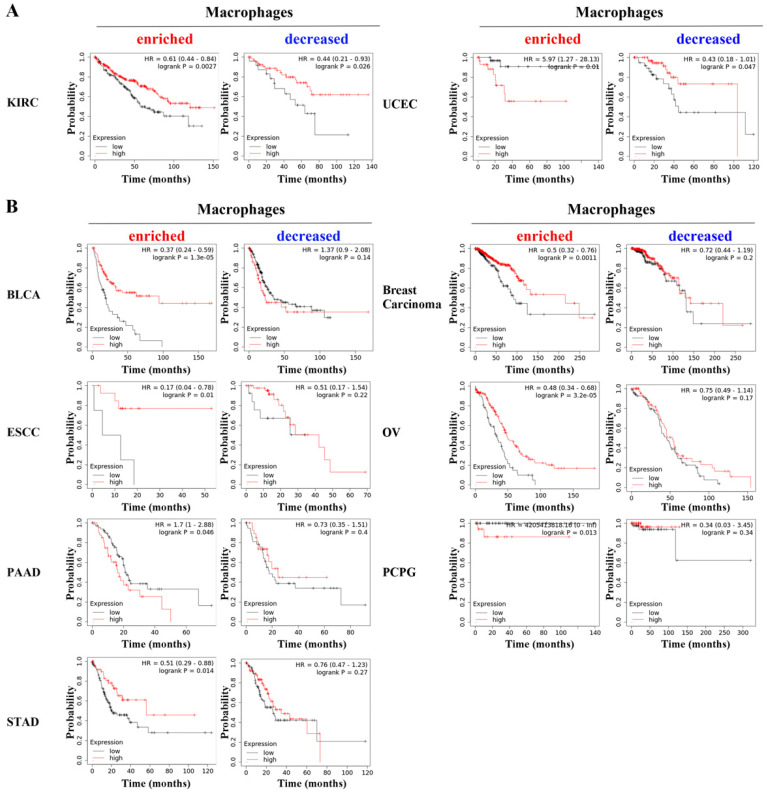
PD-L1 showed statistically significant prognostic value in KIRC and UCEC (A) based on the specific analysis of groups with enriched or decreased macrophage levels, while it did not show prognostic value in other seven types of cancer (B).

We further performed specific analysis based on enriched macrophages. PD-L1 showed significant prognostic value in bladder carcinoma (BLCA, HR=0.37, p=1.3e-05), breast carcinoma (HR=0.5, p=0.0011), esophageal squamous cell carcinoma (ESCC, HR=0.17, p=0.01), OV (HR=0.48, p=3.2e-05), PAAD (HR=1.7, p=0.046), pheochromocytoma and paraganglioma (PCPG, p=0.013), STAD (HR=0.51, p=0.014), KIRC (HR=0.61, p=0.0027) and UCEC (HR=0.44, p=0.026). Notably, the specific analysis based on decreased macrophages revealed that PD-L1 was still associated with the overall survival of KIRC and UCEC (Figure 2A) but was not associated with overall survival in the other seven types of cancer (Figure 2B). These results indicate that the prognostic value of PD-L1 is related to the content of macrophages in cancers.

### PD-L1 expression is correlated with macrophage infiltration levels in cancers

**Figure 3:**
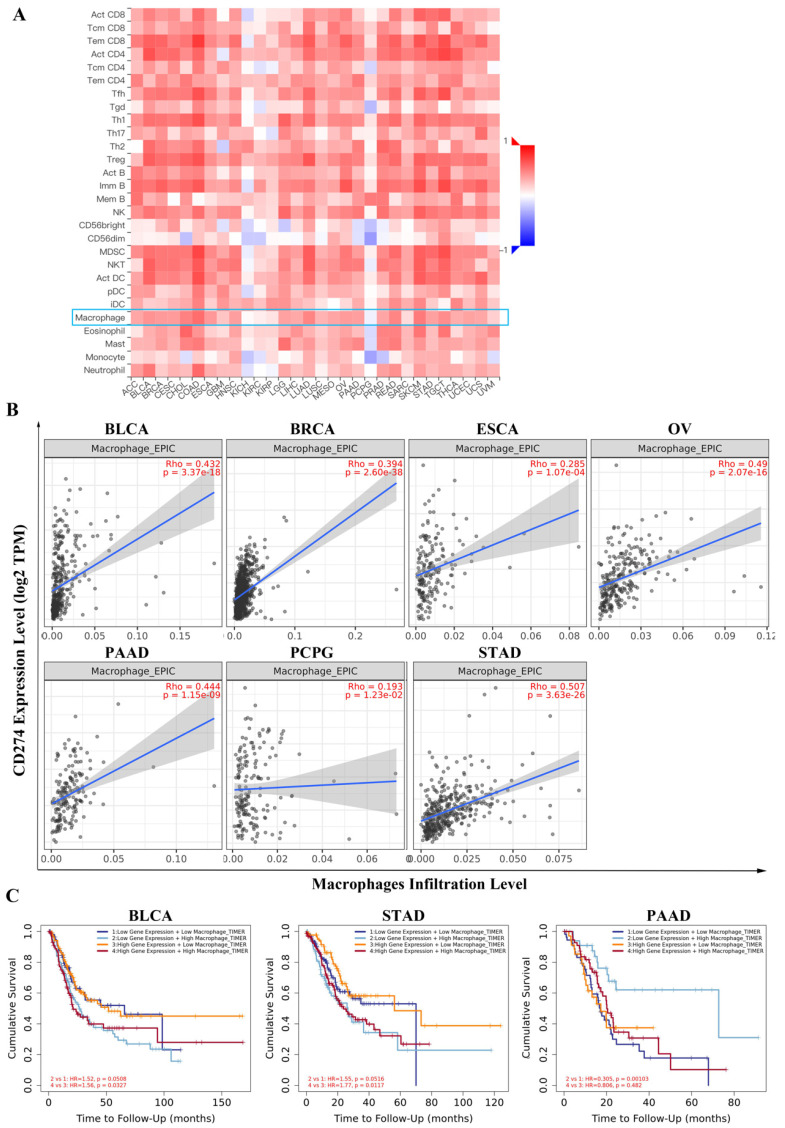
The relationship between PD-L1 expression and tumor-infiltrating lymphocyte abundance based on TISIDB (A). PD-L1 expression was correlated with the macrophage infiltration level in certain cancers based on TIMER (B). Combined consideration of PD-L1 expression and macrophage infiltration level showed prognostic value based on TIMER analysis (C).

With TISIDB, we assessed the relationship between PD-L1 expression and tumor-infiltrating lymphocyte abundance in cancers. The results showed that PD-L1 was universally associated with macrophage abundance in 30 cancer types (Figure 3A). We focused on the seven types of tumors mentioned above, and the correlations between PD-L1 expression and macrophages was analyzed with Spearman. The results showed that PD-L1 expression was significantly correlated with macrophage infiltration levels in BLCA (P < 2.2e-16), BRCA (P < 2.2e-16), ESCA (P = 1.83e-08), OA (P = 1.34e-12), PAAD (P = 1.63e-07) and STAD (P = 5.56e-15), except in PCPG (P = 0.433) (Figure S2A).

In addition, we employed the TIMER database to confirm the correlation between PD-L1 and macrophage infiltration level. As shown in Figure 3B, the PD-L1 expression level had a strong positive correlation with the level of infiltrating macrophages in BLCA (r = 0.432, P =3.37e-18), BRCA (r = 0.394, P =2.60e-38), ESCA (r= 0.285, P = 1.07e-04), OV (r = 0.49, P = 2.07e-16), PAAD (r = 0.444, P = 1.15e-09), PCPG (r = 0.193, P = 1.23e-02) and STAD (r =0.507, P = 3.63e-26). Notably, the PD-L1 expression level also influenced the prognostic value of macrophages. In the high PD-L1 expression group, but not in the low PD-L1 expression group of BLCA (HR = 1.56, P = 0.0327) and STAD (HR = 1.77, P = 0.0117), macrophages were revealed to be negatively associated with overall survival. Additionally, the expression of PD-L1 and macrophage infiltration levels showed comprehensive prognostic value in PAAD (HR = 0.305, P = 0.00103) (Figure 3C).

To validate the relationship between PD-L1 and macrophages, we further determined the correlation between PD-L1 and the marker genes of macrophages with the TIMER database. Specifically, we showed that the macrophage markers CD68 (BLCA: P =5.42e-42, BRCA: P =3.57e-104, ESCA: P =2.49e-03, OV: P =2.69e-30, PAAD: P =3.28e-14, PCPG: P =1.27e-06, STAD: P =1.27e-19), S100A9 (BLCA: P =5.91e-08, BRCA: P =3.02e-20, ESCA: P =5.48e-04, OV: P =4.16e-02, PAAD: P =4.91e-06, PCPG: P =2.26e-02, STAD: P =6.01e-08) and CSF1R (BLCA: P =4.68e-35, BRCA: P =5.68e-79, ESCA: P =2.27e-07, OV: P =2.15e-19, PAAD: P =4.1e-20, PCPG: P =1.85e-04, STAD: P =2.09e-28) were significantly associated with PD-L1 expression in the above seven types of cancer (Figure S2B). These findings suggest that there is a close relationship between PD-L1 and macrophages in tumors.

### PD-L1 is universally expressed on TAMs based on scRNA-seq data

**Figure 4:**
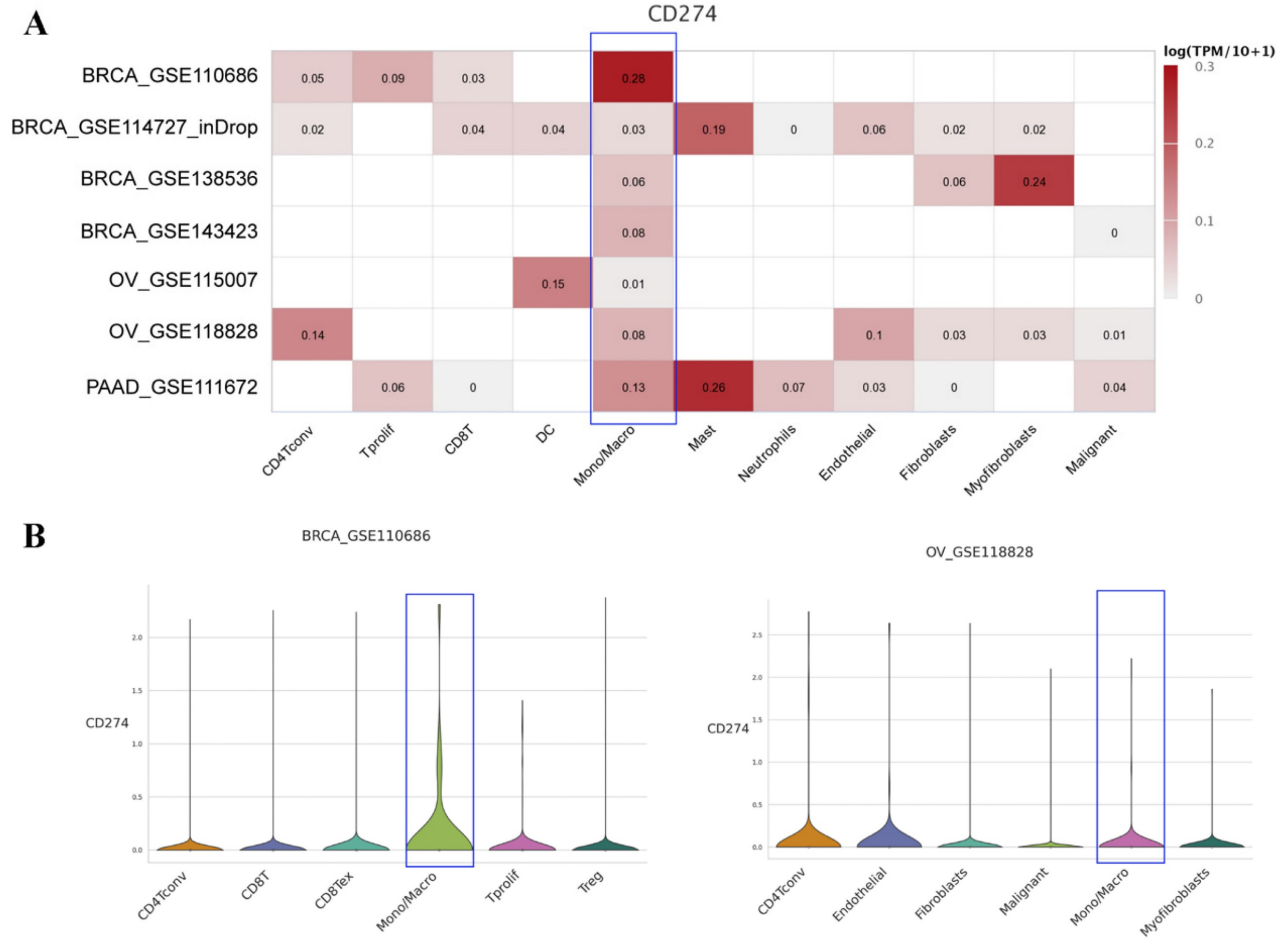
Average expression of PD-L1 in different cell types across some human cancer datasets (heatmap) based on the TISCH web portal (A). Distribution of PD-L1 expression in different cell types across the BRCA-GSE110686 dataset (B) and OV-GSE118828 dataset (C) in the form of violin plots.

To further explore the expression level of PD-L1 on macrophages, we analyzed the scRNA-seq data of different cancers with TISCH. The results showed that PD-L1 was widely expressed on macrophages in various cancers (Figure S3). We then mainly focused on the above seven types of cancers, as shown in Figure 4A. PD-L1 was found to be expressed on macrophage clusters based on the scRNA-seq data of BRCA, OV and PAAD. Specifically, in the BRCA-GSE110686 dataset, the expression of PD-L1 on the macrophage cluster was higher than that on other cell clusters. The distribution of PD-L1 gene expression in different cell types across the OV-GSE118828 dataset is also shown in Figure 4B.

### PD-L1 expression on TAMs correlates with treatment response in cancers

**Figure 5:**
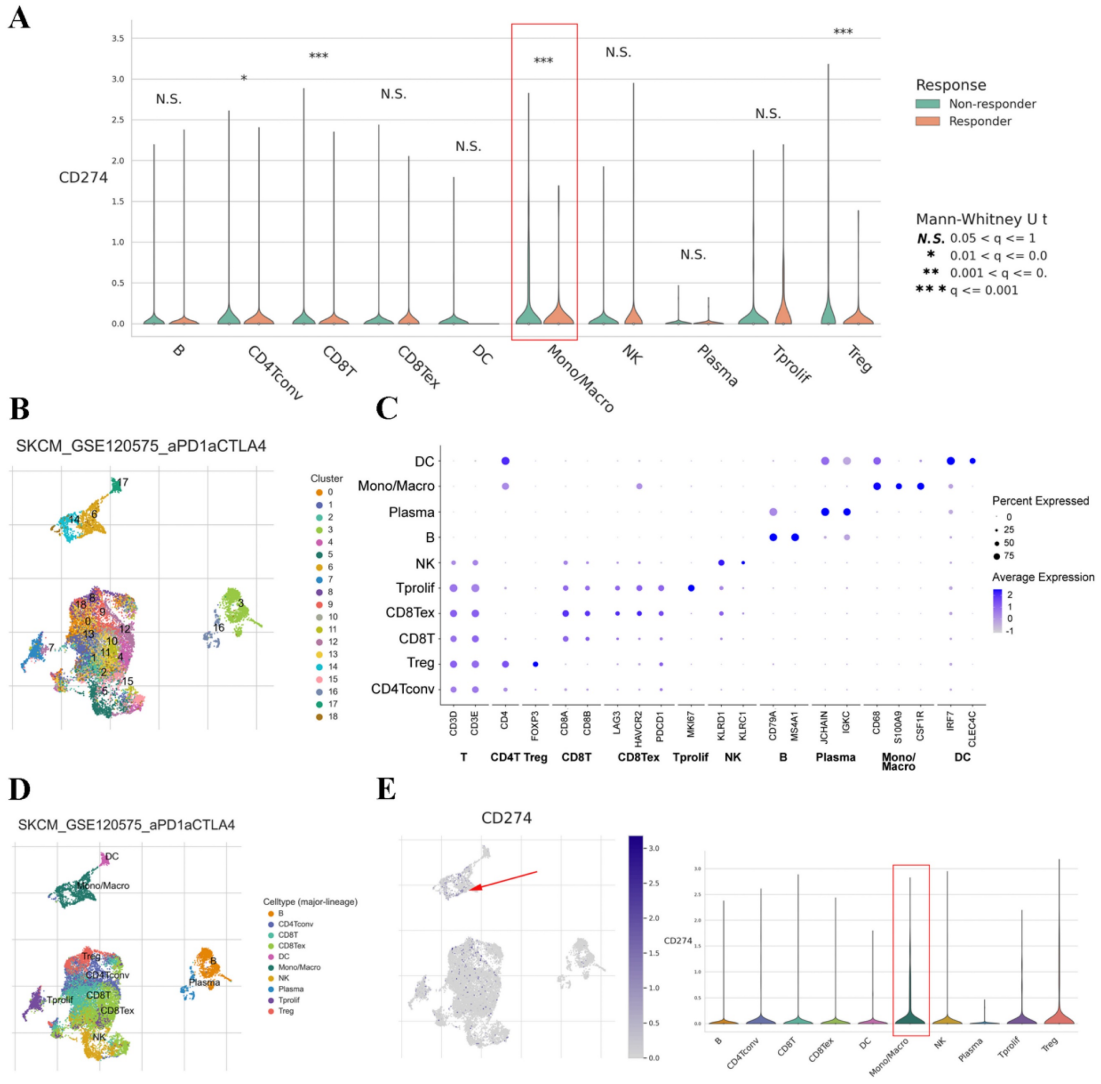
PD-L1 gene expression in different cell types grouped by treatment response based on the SKCM-GSE120575 dataset (A). tSNE plot depicting single cells (B). Marker gene expression according to cell type (major lineage) (C). Subjects are color coded according to the cell type (major lineage) on the tSNE (D). Distribution of PD-L1 expression in different cell types (E).

The TISCH dataset included limited treatment information. Thus, we also analyzed the correlation between PD-L1 expression on TAMs and the therapeutic effect in cancer patients. The results showed that the expression of PD-L1 on TAMs was significantly associated with the treatment response in SKCM and basal cell carcinoma (BCC) (Figure 5A and Figure S4A). Clustering of cells based on the SKCM-GSE120575 dataset created a detailed map comprising 18 transcriptionally homogeneous subpopulations (Figure 5B). After adjusting for cell type (major lineage) based on marker gene expression (Figure 5C and D), the expression of PD-L1 on macrophages was significantly higher than that on other cells (Figure 5E). Based on the BCC-GSE123813 dataset, PD-L1 was found to be mainly expressed on macrophages (Figure S4B and C). These results indicated that PD-L1 was highly expressed on macrophages and that its expression was associated with immunotherapy response.

## Discussion

With the in-depth study of the tumor immune microenvironment, it has been recognized that the immune escape of tumor cells is the key reason for tumor progression, and its molecular mechanism has also become one of the key topics in tumor immunotherapy research 24. Recent studies have shown that PD-L1 (CD274) and PD-1 are closely related to immune evasion and tumorigenesis 25. PD-L1 is highly expressed in a variety of tumor cells and affects the clinical outcomes of immunotherapy 26. In this study, we found that PD-L1 was upregulated to different degrees in CESC, CHOL, ESCA, HNSC, STAD, DLBC and THYM and downregulated in BRCA, LIHC, LUAD, LUSC, PRAD and UCEC. Based on these results, PD-L1 may be a good potential diagnostic biomarker for different cancers.

Studies have shown that the expression level of PD-L1 is closely related to the prognosis of tumor patients 27, which was also observed in this study. Notably, our results demonstrated that the prognostic value of PD-L1 was different between groups with enriched and decreased macrophage levels in BLCA, BRCA, ESCA, OV, PAAD, PCPG and STAD, indicating that PD-L1 may influence tumorigenesis partly through macrophages. It is well recognized that macrophages are important immune components in the tumor microenvironment 28. In our study, we further validated the significant correlations of PD-L1 with macrophage infiltration and with the marker genes of macrophages in the above seven types of cancers. Consistent with these findings, studies have revealed that macrophages can increase the expression of PD-L1 on tumor cells in ESCA and STAD 29, 30. Moreover, breast cancer cells upregulate PD-L1 expression on macrophages and promote macrophage-associated immunosuppression 31. Interestingly, circulating sPD-L1 in the blood was found to be positively associated with PD-L1+ macrophages in the blood of OV patients 32.

Based on the scRNA-seq data in TISCH, we further found that PD-L1 was widely expressed on macrophages in cancers. It has been confirmed that TAMs with high expression of PD-L1 play an immunosuppressive role in the tumor microenvironment 33. In hepatocellular carcinoma, the infiltration of PD-L1+ macrophages was negatively correlated with overall survival and disease-free survival 34. In non-small-cell lung cancer (NSCLC), the 5-year overall survival of patients with low expression of PD-L1 in macrophages was significantly higher than that of patients with high expression of PD-L1 in macrophages 35. Notably, a previous study indicated that macrophage polarization affects the antitumor efficacy of checkpoint inhibitor therapy 36. In this study, we found that the expression of PD-L1 on macrophages was significantly associated with the checkpoint inhibitor treatment response in SKCM and BCC. Due to the limited treatment information in the single-cell sequencing database, we did not perform a similar analysis in the other tumor types, but such analyses will be performed in future research.

In conclusion, our study found that PD-L1 is dysregulated in various cancers and is associated with prognosis; in addition, the relationship of PD-L1 with prognosis is partly influenced by macrophage infiltration levels. The correlation between PD-L1 and macrophages is widely observed in tumors. Moreover, PD-L1 is universally expressed on macrophages and significantly associated with the treatment response in some cancers.

## Supplementary Material

Supplementary figures.

## Abbreviations

PD-L1: programmed death receptor-1 ligand
PD-1: programmed death receptor-1
GM-CSF: granulocyte-macrophage colony-stimulating factor
CSF1: colony-stimulating factor 1
TIMER: Tumor IMmune Estimation Resource
TISCH: Tumor Immune Single Cell Hub
TAMs: tumor-associated macrophages
scRNA-seq: single-cell RNA sequencing
GEPIA: Gene Expression Profiling Interactive Analysis
CESC: cervical squamous cell carcinoma
CHOL: cholangiocarcinoma
ESCA: esophageal carcinoma
HNSC: head and neck squamous cell carcinoma
STAD: stomach adenocarcinoma
BRCA: breast invasive carcinoma
LIHC: liver hepatocellular carcinoma
LUAD: lung adenocarcinoma
LUSC: lung squamous cell carcinoma
PRAD: prostate adenocarcinoma
UCEC: uterine corpus endometrial carcinoma
DLBC: diffuse large B-cell lymphoma
GBM: glioblastoma multiforme
LGG: brain lower grade glioma
SKCM: skin cutaneous melanoma
TGCT: testicular germ cell tumor
THYM: thymoma
OV: ovarian cancer
PAAD: pancreatic ductal adenocarcinoma
BLCA: bladder carcinoma
ESCC: esophageal squamous cell carcinoma
PCPG: pheochromocytoma and paraganglioma
BCC: basal cell carcinoma
NSCLC: non-small-cell lung cancer
