# Supplementary material for: Expression of programmed death receptor-1 ligand (PD-L1) in human cancer is of prognostic value and associated with macrophage infiltration
Source: J Cancer. 2024 Nov 4;15(20):6798–807. doi: 10.7150/jca.99781 (PMC11632976; doi:10.7150/jca.99781)
Supplement: Supplementary file 1 — Supplementary figures. [file jcav15p6798s1.pdf]

Supporting Information

Expression of programmed death receptor-1 ligand (PD-L1) in human cancer is of prognostic value and associated with macrophage infiltration

Yu Liu[a,b,†], Qian Li[c,†], Gangchi Cao[d,†], Huijuan Wei[e], Chuansong Xue[a], Jianhao Liu[a,*]

a Sanya Hospital of Traditional Chinese Medicine, Sanya, China

b Department of Medical Oncology, Hangzhou Cancer Hospital, Hangzhou, China

c Department of Hematology & Oncology, Shanghai Children's Medical Center, Shanghai Jiao Tong University School of Medicine, National Health Committee Key Laboratory of Pediatric Hematology & Oncology, Shanghai, China.

d Department of Clinical Medicine, Sun Yat-sen University School of Medicine, Guangzhou, China

e Guangzhou University of Chinese Medicine, Guangzhou, China

E-mail: Ljhamdoctor08@yeah.net (Jianhao Liu)

† These authors have contributed equally to this work.

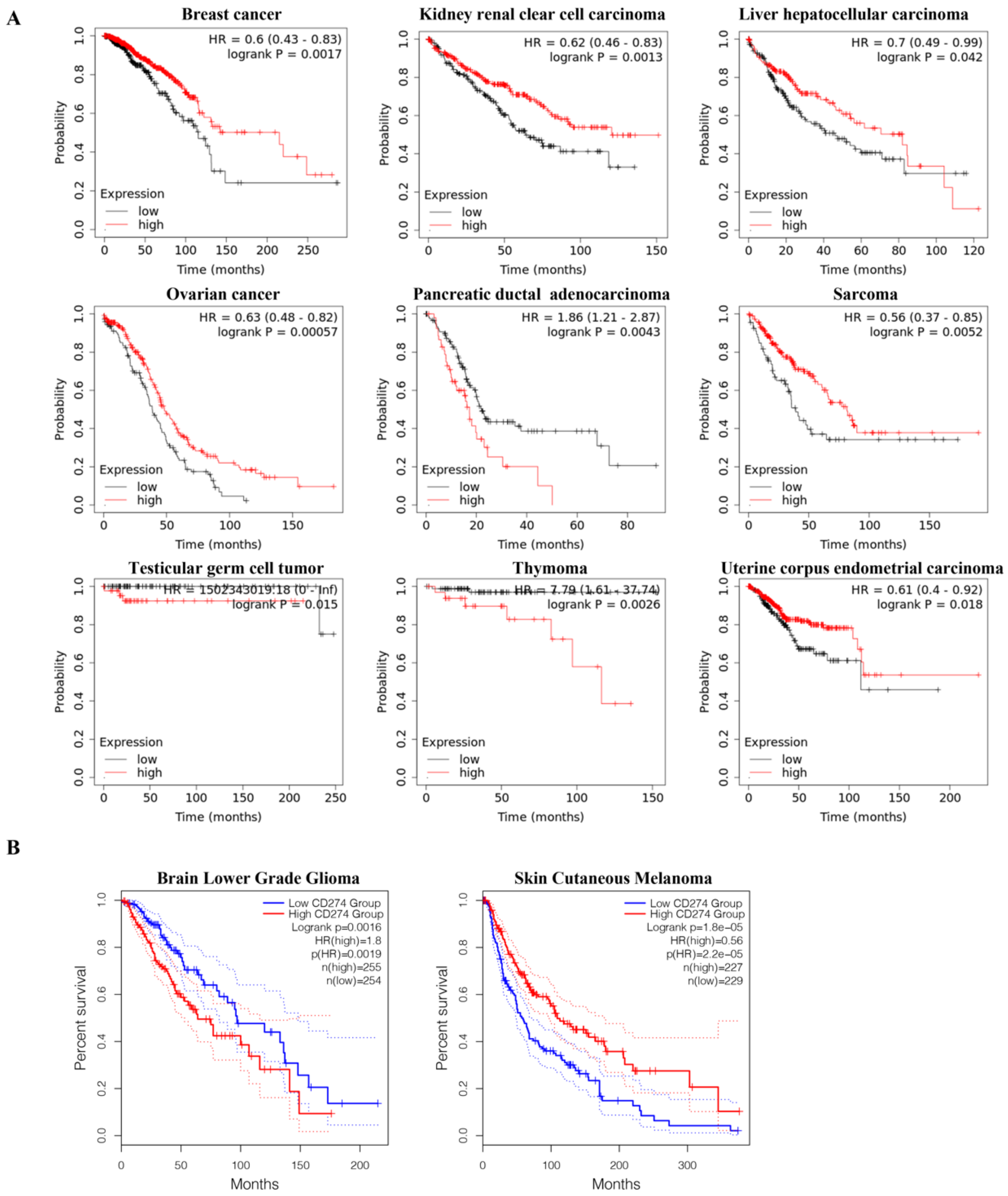

Figure S1. Statistically significant correlation between PD-L1 expression and the overall survival of cancer patients identified based on the Kaplan-Meier plotter database (A) and GEPIA (B).

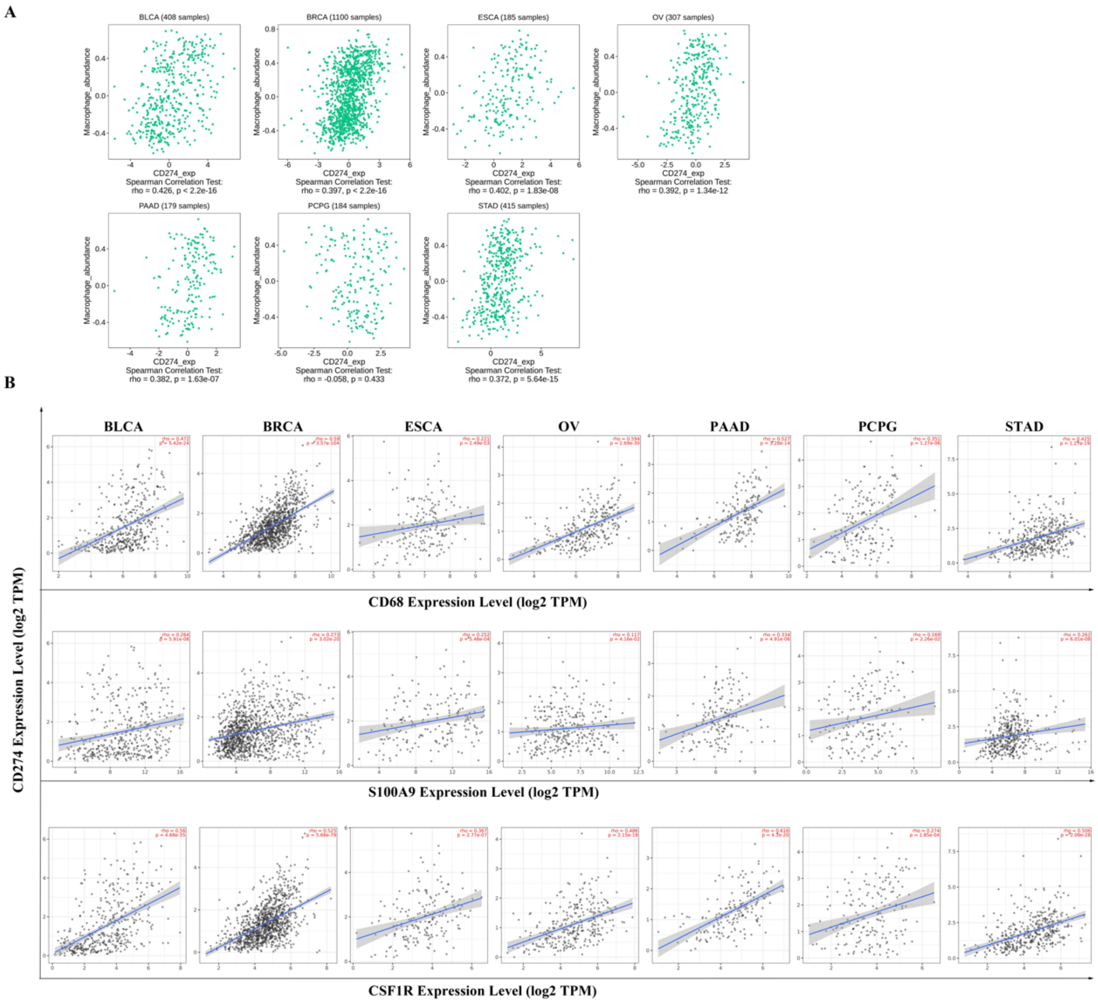

Figure S2. PD-L1 expression was correlated with the macrophage infiltration level in some cancers based on TISIDB data (A). The associations between PD-L1 and macrophage marker genes based on TIMER data (B).

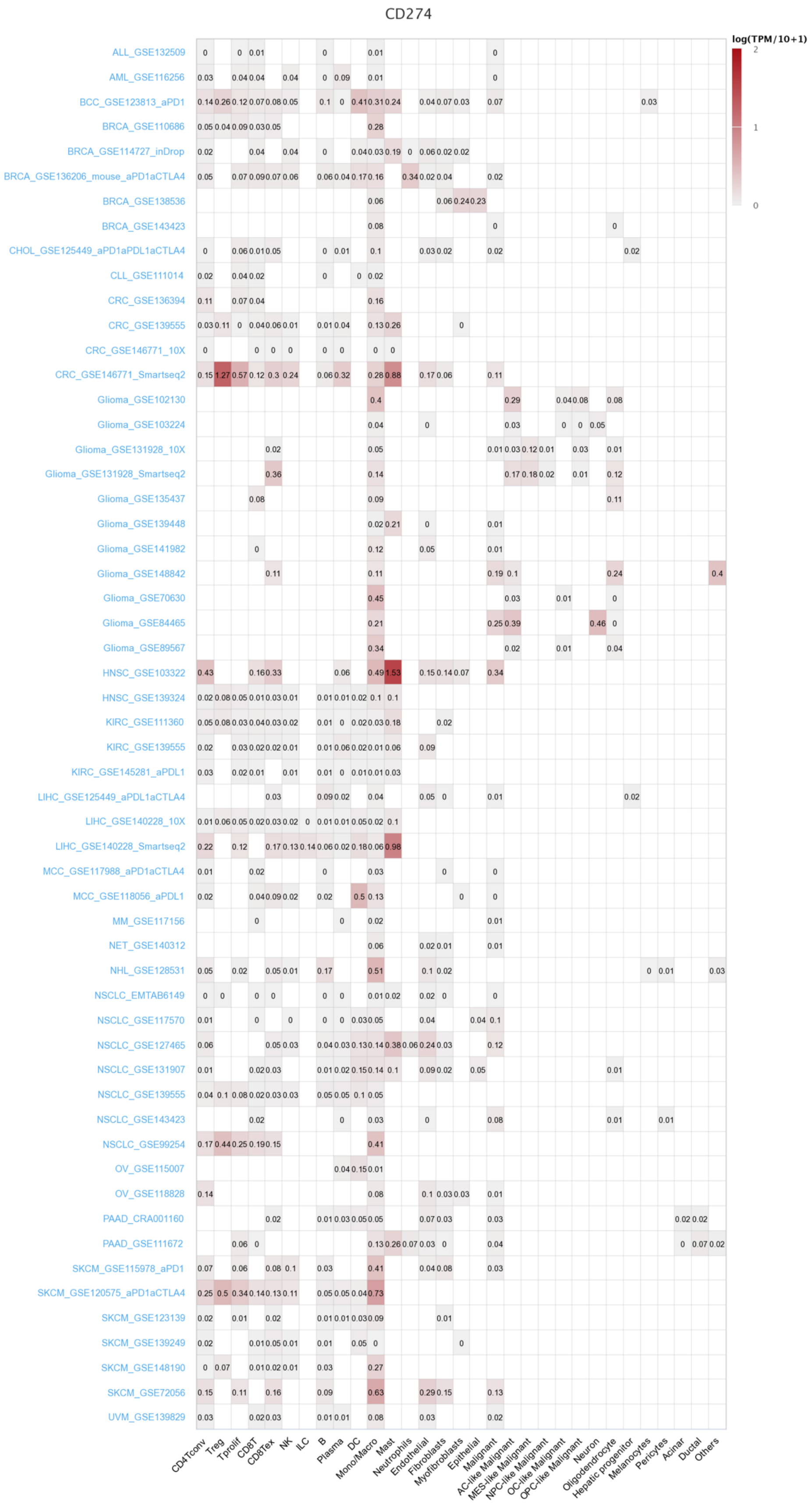

Figure S3. Average expression of PD-L1 in different cell types across human cancer datasets (heatmap) from the TISCH web portal.

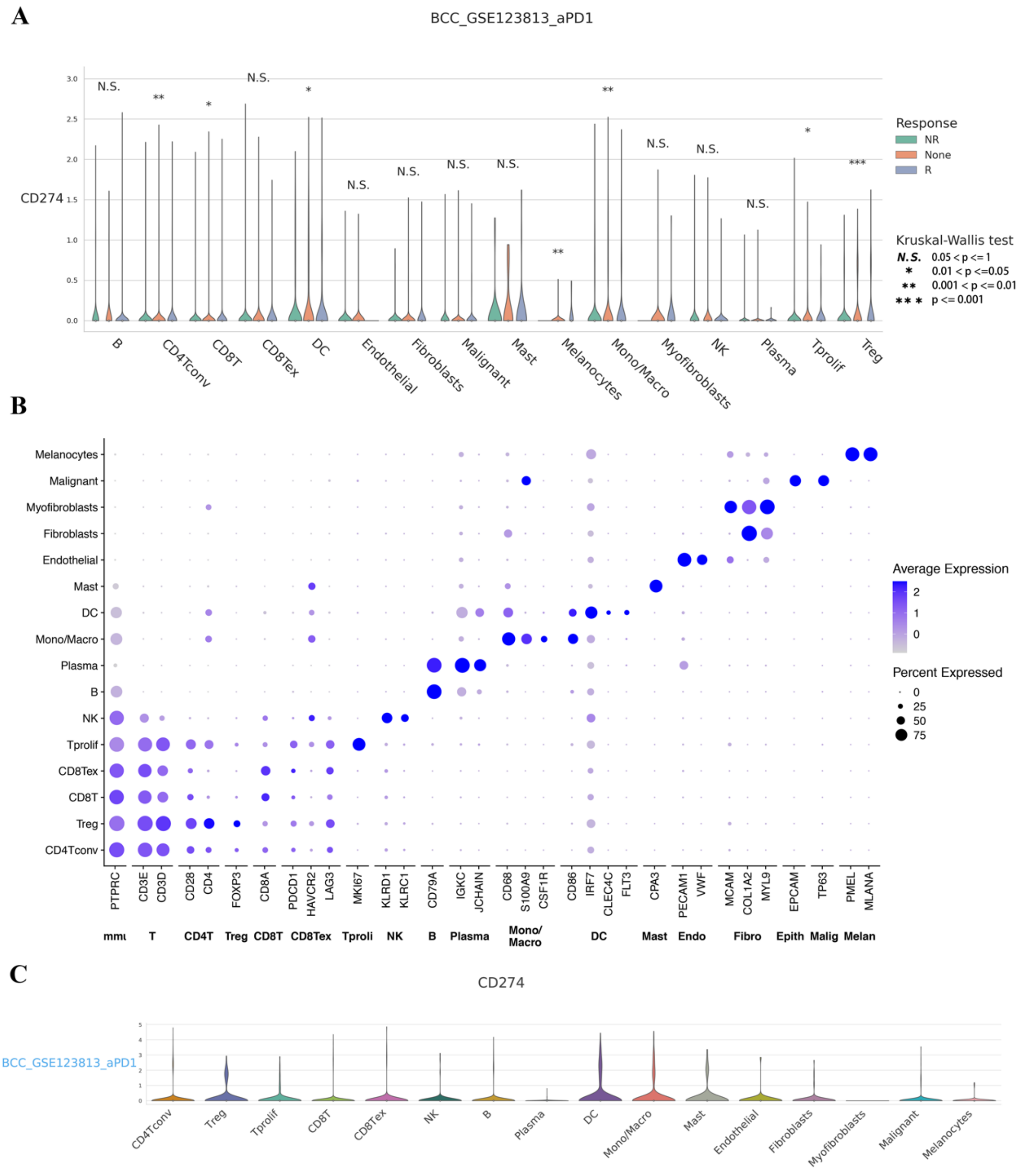

Figure S4. PD-L1 gene expression in different cell types grouped by treatment response based on the BCC-GSE123813 dataset (A). Marker gene expression according to cell type (major lineage) (B). Distribution of PD-L1 expression across different cell types in the form of violin plots (C).
